# 

**Published:** 1894-07-28

**Authors:** 


### CONTENTS.

The Chelsea Hospital for Women - - - - - - - 349

Around the Hospitals - - - - - - - - - - 350

The Ethylates, or Caustic Alcohols - - - - - - 351

Dr. Pavy's Researches on Diabetes - - - - - - 352

Notes and News - - - - - - - - - - - 354

Medical Progress and Hospital Clinics.—

Chronic Catarrh of the Middle Ear - - - - - - 355

Medical Progress.—Diseases of Children - - - - - 357

Progress in Surgery.—Bone, Joint, and Orthopædic Surgery - 358

General Surgery - - - - - - - - - - 359

New Appliances and Things Medical - - - - - - 360

Annotations. — Physiology and Collectivism — Mr. Gladstone's Sight—Barrack Children: An Object Lesson - - - - 353

The Institutional Workshop:

Practical Departments - - - - - - - - - 363

Hospital Festivals and Meetings - - - - - - - 363

A Lunatic's Relatives - - - - - - - - - 363

Chelsea Hospital for Women - - - - - - - - 364

Scraps and Gleanings - - - - - - - - - 364

The Book World of Medicine and Science - - - - 365

The Student of Medicine - - - - - - - - - 365

### EXTRA SUPPLEMENT.—THE NURSING MIRROR.

News from the Nursing World.—Registration of Midwives—Employment of Epileptics—A Pleasant Day—Nurse Dolls at Stamford—Night Nursing—Massage—Queen's Nurses at Drogheda—Lepers in Norway—Male Nurses—Nurses in France—Madagascar—Little Nourishment—Short Items - - - - - - - - - - - - clxxii
On General Nursing.—XXII. Typhoid Fever - - - - clxxii
Suggestions for Intending Probationers - - - - clxxiv
Chester Workhouse Infirmary - - - - - - - clxxiv
Miss Hampton in England - - - - - - - - clxxv
The Plague in Hong Kong - - - - - - - - clxxvi
Hints on Disinfection - - - - - - - - - clxxvii
Entertainments - - - - - - - - - - - clxxvii
Reserve of Nurses - - - - - - - - - - clxxvii
Wants and Workers - - - - - - - - - - clxxvii
Home and Hospital for Jewish Incurables, Victoria Park Road - - - - - - - - - - - clxxviii
Everybody's Opinion - - - - - - - - - - clxxix
Appointments - - - - - - - - - - - - clxxx
Nursing in Germany - - - - - - - - - - clxxx
Minor Appointments - - - - - - - - - - clxxx
Notes and Queries - - - - - - - - - - - clxxx

